# Research on Face Recognition and Privacy in China—Based on Social Cognition and Cultural Psychology

**DOI:** 10.3389/fpsyg.2021.809736

**Published:** 2021-12-24

**Authors:** Tao Liu, Bijiao Yang, Yanan Geng, Sumin Du

**Affiliations:** Department of Sociology, Hangzhou Dianzi University, Hangzhou, China

**Keywords:** face recognition, technology acceptance model, social cognitive, cross-culture, privacy concerns psychology, perceived risk, trust, cultural psychology

## Abstract

With the development of big data technology, the privacy concerns of face recognition have become the most critical social issue in the era of information sharing. Based on the perceived ease of use, perceived usefulness, social cognition, and cross-cultural aspects, this study analyses the privacy of face recognition and influencing factors. The study collected 518 questionnaires through the Internet, SPSS 25.0 was used to analyze the questionnaire data as well as evaluate the reliability of the data, and Cronbach’s alpha (α coefficient) was used to measure the data in this study. Our findings demonstrate that when users perceive the risk of their private information being disclosed through face recognition, they have greater privacy concerns. However, most users will still choose to provide personal information in exchange for the services and applications they need. Trust in technology and platforms can reduce users’ intention to put up guards against them. Users believe that face recognition platforms can create secure conditions for the use of face recognition technology, thus exhibiting a higher tendency to use such technology. Although perceived ease of use has no significant positive impact on the actual use of face recognition due to other external factors, such as accuracy and technology maturity, perceived usefulness still has a significant positive impact on the actual use of face recognition. These results enrich the literature on the application behavior of face recognition and play an important role in making better use of face recognition by social individuals, which not only facilitates their daily life but also does not disclose personal privacy information.

## Introduction

Face recognition is a biometric recognition technology that uses pattern matching to recognize individual identities based on facial feature data. Compared to traditional non-biological recognition and physiological feature recognition technology, face recognition technology has specific technical advantage (Jiang, 2019). Nowadays, relying on ubiquitous mobile camera devices, face recognition technology has been widely used in various fields, including face attendance, face payment, smart campus, access control system, and security system, which demonstrate the advances in the face recognition service level in the intelligent hardware system. Face recognition technology has dramatically improved the intelligence level of business systems in these fields. The human face is rich in features. In the society of acquaintances in the past, the face was the foundation for us to involve emotional communication and social relations with others.

Technology has been one of the most important factors that changed the way of life and commercial activities of human society. With continuous innovation and the development of technology, human society is changing rapidly. Technological innovation has changed people’s lifestyles in the spheres of shopping, education, medical services, business organizations, and so on. “Technology is not only an essential tool for finding out new ways to join different actors in service innovation processes, but also as an element able to foster the emergence of new and ongoing innovations” (Ciasullo et al., 2017). For example, in the healthcare service ecosystem, health care providers adapt to the innovative medical service ecosystem so that patients can obtain better medical services. Medical service innovation has had a great impact on the continuous reconstruction of the service ecosystem (Ciasullo et al., 2017). Technology forces the market to change constantly, and the changing market leads business organizations to innovate. “The contemporary world is characterized by a fast changing environment. Business organizations are faced with the challenge of keeping pace with developments in the field of technology, markets, cultural and socio-economic structures” (Kaur et al., 2019). In the era of big data and information, business organizations must “to explore how cognitive computing technology can act as potential enabler of knowledge integration-based collaborations with global strategic partnerships as a special case” (Kaur et al., 2019).

At present, innovations in network technology provide the greatest convenience and advantages for organizations dealing with such networks. “Small and medium-sized enterprises (SMEs) have been considered the most innovative oriented businesses in developed countries even in emerging markets acting as pioneer in the digital transformational word.” Meanwhile, it is important for technology upgrading, knowledge spillover, and technology transfer to explore SMEs’ competitiveness (Del Giudice et al., 2019).

Knowledge and technology transfer is a “pathway” for accelerating economic system growth and advancement. Technology transfer can be explored from theory to practice for knowledge and technology. From the users’ perspective, technology transfer affects their sense of use and experience (Elias et al., 2017). Big data analytics capabilities (BDAC) represent critical tools for business competitiveness in highly dynamic markets. BDAC has both direct and indirect positive effects on business model innovation (BMI), and they influence strategic company logics and objectives (Ciampi et al., 2021). “In the world of Big Data, innovation, technology transfer, collaborative approaches, and the contribution of human resources have a direct impact on a company’s economic performance.” Therefore, big data companies should make corresponding changes in management and strategy. Moreover, skilled human resources have a positive contribution to the company’s economic performance. “Information and knowledge are the foundation on which act for aligning company’s strategies to market expectations and needs” (Caputo et al., 2020).

With the arrival of the era of artificial intelligence, intelligent social life has become a reality, and artificial intelligence has become a new engine for China’s economic and social development. According to the latest data released by the China Internet Information Center, the number of artificial intelligence enterprises in China ranks second in the world (CNNIC, 2020). As a new technology, face recognition—a typical application of artificial intelligence—rises with the construction of a smart city According to the statistics presented in the *Report on In-depth Market Research and Future Development Trend of China’s Face Recognition Industry* (2018–2024) released by the Intelligence Research Group, it is estimated that the face recognition industry in China will reach 5.316 billion Yuan by 2021 (Biometrics Identity Standardization [BIS], 2020). As the gateway connecting humans and intelligence face recognition has excellent development potential.

Given that the modern era emphasizes looks, the face remains socially functional, but technology has given it new meaning and a mission. The attributes and features of a facial image are enough to convey a person’s identity. When our face is tied to our personal information and even used as a password substitute, it is no longer the traditional concept of face. Face recognition technology can extract personally identifiable information, such as age, gender, and race, from images. To some extent, in the Internet age, almost everyone’s personal information is displayed without any protection.

With the technical support of big data, user portraits based on facial recognition and a variety of personal data have increasingly become identification for individuals in this day and age (Guo, 2020). From face-swapping apps, access by face recognition to Hangzhou Safari Park, the application of face recognition in subway security checks, to the formulation of the Personal Information Protection Law of the People’s Republic of China (PRC), a series of public opinions have brought face recognition to the forefront. On the other hand, Internet privacy, which has been neglected so far, is increasingly taken seriously by the public.

The issues of face recognition and privacy have been studied extensively by experts and scholars in their respective fields, but there are few empirical studies on the combination of the use of face recognition and personal privacy security. At present, most scholars’ research on face recognition focuses on face recognition algorithms, recognition systems, legal supervision and security, users’ willingness to accept face payments, and the application of face recognition in the library. No quantitative research has been conducted on the relationship between the use of face recognition technology and people’s attitudes toward privacy issues. Therefore, based on the two main determinants of the technology acceptance model (TAM) and according to public attitudes toward privacy and the specific context of the use of face recognition in the current networked environment, variables such as privacy concerns, risk perception, and trust are introduced in this study to build the hypothesis model of the actual use of face recognition. The concept of privacy concerns is applied to the research on personal information security behavior of facial recognition users, which further expands the practical scope of the privacy theory and provides suggestions to promote the development of facial recognition applications.

This research makes two contributions. First, it demonstrates the impact of privacy concerns, perceived risk, trust, social cognition, and cross-cultural aspects on facial recognition. This result enriches face recognition literature, and a hypothesis model based on perceived ease of use and perceived usefulness—the two determinants of user behavior—is created. Second, this research confirms that the privacy paradox still exists. In the digital information age, most users will still choose to provide personal information in exchange for the services and applications they need. Trust, social cognition, and culture play a vital role in intelligent societies and virtual interactions. Meanwhile, when technology applications can provide users with diversified and user-friendly functions, their perceived usefulness is significantly improved.

The structure of the article is as follows. In section “Theoretical Basis and Research Hypothesis,” we examine the theoretical basis and research hypothesis. Section “Variable Measurement and Data Collection” describes variable measurement and data collection, including questionnaire design and data collection. Section “Data Analysis” presents the results of the data analysis. Section “Conclusion” discusses the key findings of the research along with the final remarks.

## Theoretical Basis and Research Hypothesis

### Privacy

In the era of mobile data services based on big data, “the nature of economic exchange is more inclined to exchange personal information for personalized services. Privacy violations may occur in the acquisition, storage, use and transaction of personal information, thus giving rise to problems in information privacy” (Chen and Cliquet, 2020). Moreover, in the Internet environment, information privacy security in intelligent society is increasingly threatened. Since facial recognition is based on the acquisition of human face image information and face information demands privacy, face information security becomes the focus of the public when choosing whether to use facial recognition technology. On the one hand, human faces are rich in features, which provide powerful biometric features for identifying individuals; thus, a third party can identify individuals through face positioning, and so it is necessary to prevent the malicious collection and abuse of such information. On the other hand, through image storage and feature extraction, a variety of demographic and private information can be obtained, such as facial age, health status, and even relatives, which leads to unnecessary privacy invasion (Zahid and Ajita, 2017). Therefore, in view of the uniqueness of human face and information privacy, the focus of this paper will be whether the public’s actual use of face recognition is affected by their attitudes toward personal privacy and the perceived risk of personal data.

### Privacy Concerns

Privacy concerns are widely used to explain the behavior intention of users (Zhang and Li, 2018). In the Internet field, privacy concerns of users include people’s perceptions and concerns about improper access, illegal acquisition, illegal analysis, illegal monitoring, illegal transmission, illegal storage, and illegal use of private information (Wang et al., 1998). Users do not have full control over the use of their personal information. Thus, users become concerned about privacy when it may be violated due to security loopholes or inappropriate use or when individuals perceive the risk of privacy infringement.

Personal privacy in the age of mobile data services involves both online and offline domains. The extensive use of various personal biological information applications poses new challenges to personal privacy security. Specifically, with the progress of computer algorithms, the Internet of Things, and other technologies, the threshold of information collection becomes increasingly lower, and computerized information may be easily copied and shared, resulting in problems such as secondary data mining and inadequate privacy (Qi and Li, 2018). In the existing research on privacy concerns, Cha found that there is a negative correlation between users’ concerns regarding the information privacy of a technology-driven platform and the frequency of users using the media (Cha, 2010). McKnight conducted research on Facebook, whereby they found that the greater the concern about privacy is in a medium, the less willing people are to continue using the medium for fear of personal information being abused (McKnight et al., 2011). In the context of big data, the privacy concerns of face recognition users originate from the risk of facial image information being collected and used without personal knowledge or consent or the risk of personal biometrics being transmitted or leaked. In other words, the cautious choice of face recognition application is influenced by the extent of individual concerns regarding privacy. Considering these notions, the following hypothesis is proposed:

Hypothesis 1: Privacy concerns have a negative impact on the actual use of face recognition.

### Perceived Risk

Due to the virtuality or uncertainty of a network, perceived risk is an individual’s perception of the risk of information breach. The perceived risk of facial recognition may arise from the disclosure or improper use of face information. Chen conducted an empirical study on this and believed that the degree of individuals’ concerns for information security is affected by the perceived network risk (Chen, 2013). Norberg et al. (2007) showed in their study that the negative effect of perceived disclosure is affected by perceived risk. In other words, the more users perceive that the disclosure of personal information will lead to the illegal breach of privacy and other adverse effects, the more they will be concerned about the security of personal privacy. Not only is the degree of privacy concerns positively affected by perceived risk, but studies have also shown that perceived risk also affects actual use behavior (Zhang and Li, 2018). Hichang’s (2010) research results show that the degree of severity of privacy risks perceived by users is positively correlated with the degree of their self-protection behaviors. When people realize that their personal information is at risk, they take active preventive actions. Therefore, in this paper, regarding the intention to use facial recognition, it is believed that the higher the risk perceived by users, the more users will pay attention to the breach of personal privacy, thus affecting the actual use of facial recognition. In this vein, the following hypotheses are proposed:

Hypothesis 2: Perceived risk has a positive effect on privacy concerns.
Hypothesis 3: Perceived risk has a negative influence on the actual use of face recognition.

### Trust Theory

Simmel (2002) pioneered the sociological study of trust, believing that trust is an essential comprehensive social force. Putnam (2001) believed that trust is an essential social capital and can improve social efficiency through actions that promote coordination and communication. In an intelligent social environment, social transactions cannot occur without trust. Hence, trust has also become an essential factor in the study of privacy issues. In the context of face recognition, trust is defined as users’ belief in the ability of face recognition technology and application platforms to protect their personal information. Joinson et al. (2010) found in his study that users’ perceived risk to personal privacy is affected by their degree of trust. Moreover, through research on the behavioral intention of intelligent media use, some scholars present that trust will directly affect the use intention, and there is a significant correlation between trust and users’ use intention. Therefore, the following hypotheses are proposed:

Hypothesis 4: Trust negatively affects the perceived risk of users with face recognition.
Hypothesis 5: Trust positively affects the actual use of face recognition.

### Technology Acceptance Model

The TAM is widely used to explain users “acceptance of new technologies and products, and it is the most influential and commonly used theory to describe individuals” degree of acceptance to information systems (Lee et al., 2003). The TAM is used for research in different fields: education (Scherer et al., 2019), hospitals and healthcare (Nasir and Yurder, 2015; Fletcher-Brown et al., 2020; Hsieh and Lai, 2020; Papa et al., 2020), sports and fitness (Lunney et al., 2016; Lee and Lee, 2018; Reyes-Mercado, 2018), fashion (Turhan, 2013; Chuah et al., 2016), consumer behavior (Wang and Sun, 2016; Yang et al., 2016; Kalantari and Rauschnabel, 2018), gender and knowledge sharing (Nguyen and Malik, 2021), wearable devices (Magni et al., 2021), human resource management (Del Giudice et al., 2021), Internet of Things (Caputo et al., 2018), technophobia and emotional intelligence influence on technology acceptance (Khasawneh, 2018).

In this study, a hypothesis model is developed based on perceived ease of use and perceived usefulness, two determinants of user behavior.

Perceived usefulness refers to the extent to which users believe that using a specific system will improve their job performance. Perceived ease of use refers to the ease with which users think a particular system can be used, which also affects their perceived usefulness of technology (Davis, 1989). The easier it is to use face recognition, the more useful it is considered be. For the purpose of this study, face recognition aims to realize multiple functions, such as providing efficient and convenient services. Therefore, the definition of perceived usefulness should be extended to users think face recognition can improve the degree of convenience and service. In this paper, the ease of using a face recognition application refers to users’ perceived ease of use of the technology. Previously, Davis (1989) conducted an empirical study on the e-mail system and concluded that perceived ease of use has a positive impact on the use of applications. In a study on the adoption and use of information systems in the workplace, Venkatesh and Davis (2000) demonstrated that perceived usefulness has a positive impact on people’s usage behavior. With the extensive application of the TAM in the information system, the face recognition technology studied in this paper also comprises intelligent media. Perceived usefulness is an important variable that affects the use of face recognition. Thus, the following hypotheses are proposed:

Hypothesis 6: Perceived ease of use has a positive impact on perceived usefulness.
Hypothesis 7: Perceived ease of use has a positive influence on the actual use of face recognition.
Hypothesis 8: Perceived usefulness has a positive impact on the actual use of face recognition.

**FIGURE 1:**
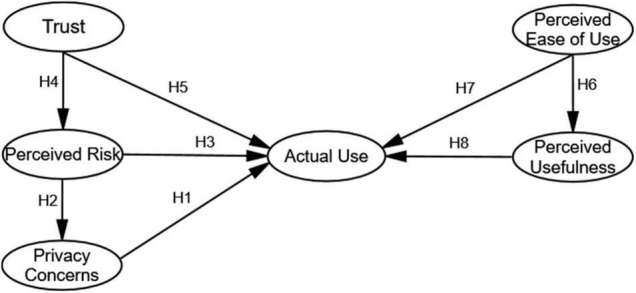
Structural equation model.

The research model of this paper is shown in Figure 1.

## Variable Measurement and Data Collection

### Questionnaire Design

In order to ensure the scientificity and credibility of the measurement variables, this study modified the mature scale in previous studies and combined it with the information concerns of current users on the use of face recognition and developed a questionnaire. This questionnaire consists of two parts. The first part investigates the demographic characteristics of users, such as gender and age. The second part is measured by a Likert scale. The options of each measurement item include “Strongly disagree,” “Disagree,” “Neither agree nor disagree,” “Agree,” and “Strongly agree.” The survey included seven latent variables and 21 measured variables. Latent variables included perceived ease of use, perceived usefulness, privacy concerns, risk perception, trust, and actual use. The contents of the scale are shown in Table 1.

**TABLE 1:** Design of measurement items for variables studied.

Factor	Measurement item	Measurement item design	Reference source
Privacy concerns	PC1	It is risky to provide face information in the big data environment	Liu, 2013
	PC2	Face recognition may result in the illegal acquisition of personal biometrics by a third party	
	PC3	Face recognition may result in the leakage or abuse of personal information and transaction information	
Perceived risk	PR1	I am worried that another person is misusing my face information	Liu, 2013; Zhang and Gong, 2018
	PR2	I am worried that face recognition has collected a lot of my personal information	
	PR3	I am worried that unidentified users can obtain my information	
Perceived ease of use	PEU1	It is easy for me to use face recognition	Davis, 1989; He and Huang, 2020
	PEU2	I do not think the operation of face recognition is complicated	
	PEU3	I think it is easy to complete face recognition	
Perceived usefulness	PU1	I feel face recognition can help me save time	Davis, 1989; Zhang and Gong, 2018
	PU2	With face recognition, I can get the service and convenience I desire	
	PU3	In general, I think face recognition is useful	
Trust	T1	I think face recognition technology is trustworthy	Qi and Li, 2018
	T2	I think the privacy protection measures of face recognition technology have been perfect	
	T3	I think the whole process of face recognition is safe	
	T4	I believe that the face recognition platform can protect my privacy	
	T5	I believe that the face recognition platform will abide by its agreement to protect my privacy	
Actual use	AU1	I would like to use face recognition	Yu, 2018
	AU2	In the case of multiple recognition methods, I will give priority to face recognition	
	AU3	I will always use face recognition	
	AU4	I hope you can use face recognition in the future	

### Data Collection

In this study, the questionnaire was designed on the survey platform^1^ and distributed in the form of links through WeChat, QQ, and other channels. The survey was conducted from May 26 to June 10, 2020, and a total of 635 questionnaires were recovered. The subjects of the questionnaire were users of face recognition technology. After the second screening, 518 valid questionnaires remained after the elimination of incomplete questionnaires and all the questionnaires with the same options. The specific statistics are shown in Table 2.

**TABLE 2:** Statistical analysis of demographic characteristics (*N* = 518).

Statistical item	Category	Frequency	Percentage%
Gender	Male	226	43.6
	Female	292	56.4
Age	18–25	383	73.9
	25–35	101	19.5
	35–45	24	4.6
	45–55	8	1.5
	55–	2	0.4
Education	Primary school and below	1	0.2
	Junior middle school	8	1.5
	Senior high school	20	3.9
	College	40	7.7
	Bachelor	342	66.0
	Master and above	106	20.5
City	First-tier and new first-tier cities	304	58.7
	Second-tier cities	79	15.3
	Third-tier cities	55	10.6
	Fourth and fifth-tier cities	59	11.4
	Unknown	21	4.1
Is face information private?	Yes	446	86.1
	No	72	13.9
Total		518	100.0

From the reported statistics, it can be seen that the gender ratio of the sample data is balanced. The age structure of the respondents is mainly between 18 and 35 years old, so it is an overall young sample, conforming to the age characteristics of the main user group of facial recognition. The respondents mostly have a high level of education, with a bachelor’s degree or above. In terms of urban distribution, 58.7% of respondents came from first-tier cities and new first-tier cities. The sample coverage is reasonable and thus representative. As for privacy, more than 86.1% of respondents believe that face information is private. Consequently, the sample data collected in this questionnaire applies to the relevant research on the privacy problems of face recognition users.

## Data Analysis

### Reliability and Validity Analysis

For this study, SPSS 25.0 was used to analyze the collected data and evaluate the reliability of the data. Cronbach’s alpha (α coefficient) was used to measure the data in this study. With 0.7 as the critical value, it is generally believed that when Cronbach’s α coefficient is greater than 0.7, the scale has considerable reliability. Based on the test results, the overall Cronbach’s α coefficients of privacy concerns, perceived risk, perceived ease of use, perceived use, trust, and actual use are between 0.876 and 0.907, all of which are greater than 0.7. This indicates that the measurement of each latent variable shows excellent internal consistency and that the questionnaire is reliable as a whole.

Structural validity refers to the corresponding relationship between measurement dimensions and measurement items. It is often used in research to analyze questionnaire items. According to the results of AMOS 24.0 for confirmatory factor analysis, the fitting index of confirmatory factor analysis in this study was X2/df = 2.722, which is less than 3, thus indicating that the fit was ideal. RMSEA = 0.058, which is less than 0.08, indicating that the model is acceptable. It is generally believed that when the fitting index of NFI, IFI, and CFI is greater than 0.9, it indicates that the model fits well; in this regard, NFI = 0.938, RFI = 0.925, IFI = 0.960, TLI = 0.951, CFI = 0.959. Therefore, the fitting index of this model conforms to the common standard, and the fitting degree of the model is proper.

Exploratory factor analysis is utilized to determine whether each measurement item converges to the corresponding factor, and the number of selected factors is determined by the number of factors whose eigenvalue exceeds 1. If the value of factor loading is greater than 0.6, it is generally considered that each latent variable corresponds to a representative subject (Gerbing and Anderson, 1988; Gefen and Straub, 2005).

As shown in Table 3, the values of factor loading of the latent variables, including privacy concerns, perceived risk, perceived ease of use, perceived usefulness, trust, and actual use, were all greater than 0.7, which shows that the corresponding topic of latent variables is highly representative.

**TABLE 3:** Factor load and variable combination reliability.

Path			Estimate	AVE	CR
PC1	< —	Privacy concerns	0.822	0.7318	0.891
PC2	< —	Privacy concerns	0.906		
PC3	< —	Privacy concerns	0.836		
PR1	< —	Perceived risk	0.87	0.7705	0.9096
PR2	< —	Perceived risk	0.867		
PR3	< —	Perceived risk	0.896		
PEU1	< —	Perceived ease of use	0.749	0.7157	0.8824
PEU2	< —	Perceived ease of use	0.879		
PEU3	< —	Perceived ease of use	0.902		
PU1	< —	Perceived usefulness	0.869	0.7162	0.8832
PU2	< —	Perceived usefulness	0.868		
PU3	< —	Perceived usefulness	0.8		
T5	< —	Trust	0.754	0.6191	0.8898
T4	< —	Trust	0.814		
T3	< —	Trust	0.844		
T2	< —	Trust	0.836		
T1	< —	Trust t	0.673		
AU1	< —	Actual use	0.737	0.6467	0.8795
AU2	< —	Actual use	0.817		
AU3	< —	Actual use	0.857		
AU4	< —	Actual use	0.801		

Combined reliability (CR) and average variance extracted (AVE) were used for the convergent validity analysis. Generally, the recommended threshold of CR is greater than 0.8 or higher (Werts et al., 1974; Nunnally and Bernstein, 1994). AVE is recommended to be above 0.5 (Fornell and Larcker, 1981). As shown in Table 3, the AVE of each latent variable was greater than 0.5, and CR was greater than 0.8, indicating that the convergence validity was ideal.

According to the results in Table 4, there was a significant correlation between actual use and privacy concerns, perceived risk, perceived ease of use, perceived usefulness, and trust (*p* < 0.001). In addition, the absolute value of the correlation coefficient corresponding to each variable was less than 0.5 and was less than the corresponding AVE square root. It indicates that there was a specific correlation between latent variables and a certain degree of differentiation among them, so the scale has an ideal level of discriminant validity.

**TABLE 4:** Correlation coefficient and AVE square root.

Correlation coefficient matrix between factors	Privacy concerns	Perceived risk	Perceived ease of use	Perceived usefulness	Trust	Actual use
Privacy concerns	0.7318					
Perceived risk	0.36***	0.7705				
Perceived ease of use	0.122***	0.127***	0.7157			
Perceived usefulness	0.056	0.065***	0.249***	0.7162		
Trust	–0.122***	–0.118***	0.029	0.101***	0.6191	
Actual use	–0.085***	–0.085***	0.141***	0.208***	0.326***	0.6467
AVE square root	0.855	0.878	0.846	0.846	0.787	0.804

***p < 0.001; The value on the diagonal is AVE (Average Variance Extracted).

### Correlation Analysis

Correlation analysis studies whether there is a correlation between variables and uses the correlation coefficient to measure the degree of closeness between variables. The three statistical correlation coefficients are the Pearson correlation coefficient, the Spearman correlation coefficient, and the Kendall correlation coefficient, of which the Pearson correlation coefficient is commonly used in questionnaire and scale studies (Qi and Li, 2018). In this study, SPSS 25.0 and Pearson’s correlation analysis were used to study whether there is a significant correlation between privacy concerns, perceived risk, perceived ease of use, perceived use, trust, and actual use in a hypothetical model to validate the validity of the research hypotheses.

Table 5 shows the means and standard deviations of privacy concerns, perceived risk, perceived ease of use, perceived usefulness, trust, and actual use and the Pearson correlation coefficient between the variables. From the mean, users had a higher perceived risk and a lower degree of trust. The results of correlation coefficient matrix showed that perceived risk and privacy concerns are significantly and positively correlated, and H2 was initially verified; privacy concerns, persistent risk, and actual use were negatively correlated (*r* = –0.158, *p* < 0.01), and the correlation degree was weak, preliminarily supporting H1 and H3. There was a positive correlation between perceived ease of use, perceived usefulness, and actual use (*p* < 0.01). Among these, perceived ease of use had a weak correlation with actual use (*r* = 0.292) and perceived usefulness showed a moderate correlation with actual use (*r* = 0.494); thus, H6, H7, and H8 were preliminarily verified. There was a significantly strong correlation between trust and actual use (*p* < 0.01, *r* = 0.608), so H5 was preliminarily verified. In addition, trust was also negatively correlated with perceived risk, due to which H4 was preliminarily verified.

**TABLE 5:** Correlation coefficient matrix and mean and standard deviation of variables.

	M	SD	Privacy concerns	Perceived risk	Perceived ease of use	Perceived usefulness	Trust	Actual use
Privacy concerns	3.986	0.708	1					
Perceived risk	4.070	0.745	0.687**	1				
Perceived ease of use	3.981	0.675	0.237**	0.244**	1			
Perceived usefulness	3.814	0.702	0.129**	0.158**	0.590**	1		
Trust	3.032	0.695	–0.228**	–0.220**	0.084	0.264**	1	
Actual use	3.180	0.743	–0.158**	–0.158**	0.292**	0.494**	0.608**	1

**Indicates a significant correlation at the 0.01 level (double tails).

### Path Analysis and Hypothesis Testing

**FIGURE 2:**
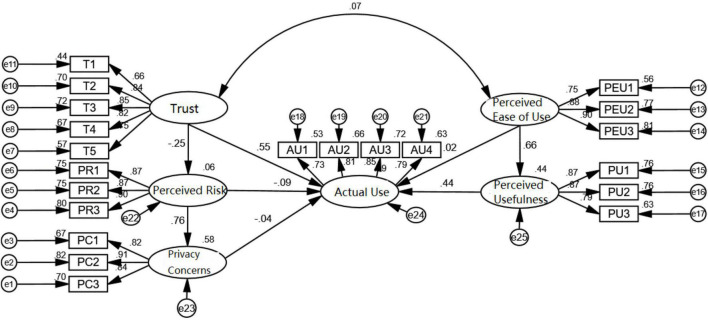
Path analysis diagram of the structural equation model.

The correlation analysis results showed that there was a correlation between the variables, so these hypotheses were preliminarily supported. Nevertheless, it could not adequately explain the systematic relationship between variables. Thus, AMOS 24.0 and the structural equation model were further employed in this study to explore the systematic relationship between the variables. As shown in Figure 2.

As can be seen from Table 6, the ratio of chi-square to the degree of freedom in the structural equation was less than 5, which is within the acceptable range. RFI, CFI, NFI, TLI, IFI, and GFI indexes were all significantly greater than 0.9, and the root mean square error of approximation (RMSEA) was less than 0.08. Thus, it shows that the structural equation model fits well.

**TABLE 6:** Fitting of the structural equation model (*N* = 518).

Fitting index	X2/df	RMSEA	GFI	AGFI	CFI	IFI	TCI	RFI	NFI
Recommended value	<3.00	<0.05	>0.90	>0.80	>0.90	>0.90	>0.90	0.9	>0.90
Acceptable value	<5.00	<0.080	>0.70	>0.70	>0.80	>0.80	>0.80	>0.80	>0.80
Actual value	3.092	0.064	0.905	0.878	0.949	0.949	0.941	0.915	0.927

According to Table 7, the hypotheses H2, H4, H5, H6, and H8 were verified, which shows that trust and perceived usefulness both positively influence the actual use intentions of face recognition users and that perceived risk also has a significant positive impact on privacy concerns. This indicates that the higher the public’s awareness of privacy is, the more risks it will perceive and the higher the public’s concerns about privacy will be. However, H1 and H3 were not accepted. From the test results, it can be seen that privacy concerns and perceived risk had a negative influence on the actual use of face recognition, but the influence was not significant. In addition, H7 was not supported, indicating that perceived ease of use had no significant influence on the actual use of face recognition.

**TABLE 7:** Results of the hypothesis test.

Hypothesis	Path	Unstandardized coefficient	Standardized coefficient	S.E.	C.R.	P	Support (Yes/No)
H1	Privacy concerns → Actual use	–0.049	–0.042	0.072	–0.679	0.497	No
H2	Perceived risk → Privacy concerns	0.693	0.759	0.042	16.699	***	Yes
H3	Perceived risk → Actual use	–0.1	–0.094	0.067	–1.5	0.134	No
H4	Trust → Perceived risk	–0.248	–0.253	0.047	–5.282	***	Yes
H5	Trust → Actual use	0.575	0.554	0.046	12.449	***	Yes
H6	Perceived ease of use→ Perceived usefulness	0.821	0.664	0.062	13.214	***	Yes
H7	Perceived ease of use→ Actual use	0.024	0.019	0.068	0.351	0.726	No
H8	Perceived usefulness→ actual use	0.443	0.435	0.057	7.727	***	Yes

***P < 0.001.

Hypotheses H1, H3, and H7 were not supported for the following reasons:

1. H1 and H3 were not supported: Perceived risk and privacy concerns had no significant adverse effect on the actual use of face recognition. It shows that the public chooses to use face recognition despite their concern and perception of privacy and risk. Some scholars have called this contradictory phenomenon a privacy paradox (Xue et al., 2016). In other words, although users are worried that face recognition may lead to improper use or disclosure of personal information, they still choose to use face recognition in the field of mobile networks. An important reason is that the application of intelligent media technology, facial recognition, is becoming increasingly prevalent in our daily lives, which is reflected in all aspects of our lives. Especially in the field of public services, relying on the digital platform has improved effectiveness and efficiency via face scanning.
2. H7 was not supported: The positive influence of perceived ease of use on the actual use of face recognition was not significant. This conclusion is not consistent with previous research, but to some extent, it confirms the correlation between perceived ease of use and the use of information systems. In other words, since ease of use involves self-efficacy cognition, technology anxiety can make users perceive it to be difficult to operate and reduce their evaluation of the ease of use of the system, thus further affecting the use of face recognition technology (Bhattacherjee, 2001). Affected by external factors such as light and image clarity, the maturity of face recognition technology is not high, and the algorithm is not accurate, which affects the public’s perceived ease of use. It also reflects that, for the face recognition technology, perceived usefulness has a more substantial impact on the actual use, and those users value the functional benefits brought by face recognition applications.

### Robustness Test of the Model

In this paper, gender, age, educational background, and city of the respondents were introduced into the model as control variables to test the robustness of the hypothesis model. The test results are shown in the figure below.

**FIGURE 3:**
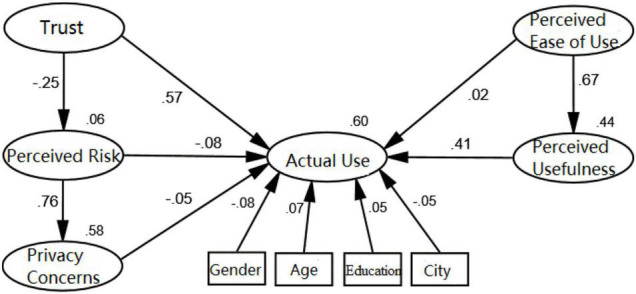
Robustness test.

It can be seen from Figure 3 that despite introducing control variables, such as gender, age, education background, and city, the relationship and significance level of each factor of the model were consistent with the conclusion of hypothesis test results above. Meanwhile, the test results of the influence of each control variable on the actual use of face recognition were not significant, indicating that the model passed the robustness test.

## Conclusion

In this study, taking the users of face recognition as the research objects, the TAM was integrated, and variables such as privacy concerns, perceived risk, and trust were added to the model to analyze the mechanism of how they affect the actual use of face recognition and explain the determinants for the use of facial recognition by the public. The results showed that the model fit well and that most of the hypotheses were supported.

Based on the results of the model analysis, this paper draws the following conclusions:

1. In the context of big data, the concept of information privacy has been continuously expanded. When users perceive the risk of their private information being disclosed through face recognition, they will have greater privacy concerns. However, although users’ privacy concerns are deep, the privacy paradox still exists. In the digital information age, most users will still choose to provide personal information in exchange for the services and applications they need.
2. Trust plays a vital role in intelligent societies and virtual interactions. In this paper, users’ trust in face recognition applications includes trust in the technology application platforms and trust in the face recognition technology itself. This study shows that the trust of technology and platform will reduce the user’s intention to safeguard themselves against it. Users believe that face recognition platforms can provide secure conditions for the use of the technology, and thus, they show a higher tendency to use such technology. On the other hand, users’ trust in face recognition technology improves, so their perceived risk of privacy information leakage is significantly reduced. In this regard, in the information age, users are willing to disclose personal information more out of their trust in face recognition technology and the related platforms.
3. In the context of face recognition as an emerging technique, the TAM still has excellent explanatory power. Although perceived ease of use has no significant positive impact on the actual use of face recognition due to other external factors, such as accuracy and technology maturity, perceived usefulness still has a significantly positive impact on the actual use of face recognition. To an extent, when technology applications can provide users with diversified and user-friendly functions, their perceived usefulness will be significantly improved.
4. The final consideration is the use management of government and technical ethics of enterprises. When developing face recognition, enterprises must pay attention to technical ethics, as well as privacy, to ensure personal privacy and protect against biological information leakage. The government must also strengthen its management of face recognition technology on a large scale to prevent enterprises and individuals from using technology to affect social security and personal privacy.

## Limitations

There are some limitations to this study. First, the sample data in the model are mostly from a young group. In future research, survey data of other age groups can be explored to discuss whether the privacy concerns of users of different age groups will affect their use of facial recognition. Second, this study focuses on the influence of privacy concerns, perceived risk, perceived ease of use, perceived usefulness, and trust on the actual use of face recognition but has not assessed whether other factors, such as user experience and usage habits, affect the actual use of face recognition. In addition, this study only analyzes the direct impact of the research variables on the actual use but fails to account for the impact of the mediating variables or moderator variables.

## Future Research Directions

Although this research provides some interesting insights, it has some significant limitations. First, future research should conduct research on different age groups to study the acceptance of face use and attention to privacy at different ages.

Second, privacy is one of the most critical ethical issues in the era of mobile data services. In the current age dominated by big data, privacy issues have become more prominent due to over-identification, technical flaws, and lagging legal construction. In this information era, the connection characteristics of the Internet pose a particularly unique information privacy threat, and many databases and records have led to the privacy boundary continually expanding. How do we balance technological enabling with privacy protection? What should users do about the privacy paradox? The different social cultures and psychology between China and the West cause people to use face recognition differently.

In terms of the impact of Western culture on face recognition, the culture pays attention to privacy and freedom, and politics and social culture affect the use of face recognition. The error and discrimination of face recognition algorithm will cause great psychological harm, coupled with the impact of social culture, and lead to social contradictions. For example, after testing the face recognition systems of Microsoft, Facebook, IBM, and other companies at MIT, it was found that the error rate of women with darker skin color is 35% higher than that of men with lighter skin color. In this regard, the algorithm was suspected to exhibit gender and racial discrimination. The algorithm is designed by people. Developers may embed their values in the algorithm, so there are artificial bias factors, which will lead to social contradictions. Therefore, politics, society, and culture have affected the governance attitude of the West. In terms of social background, religious contradictions and ethnic contradictions in Western society have intensified, and ethnic minorities have been discriminated against for a long time. The West is highly sensitive to prejudices caused by differences in religious beliefs, ethnic groups, and gender. Culturally and psychologically, the West attaches great importance to personal privacy and absolute freedom. Europeans regard privacy as dignity, and Americans regard privacy as freedom. These are some of the new problems we should focus on resolving now.

The core element of cognitive science is cognition, which is also known as information processing. Cognitive science and artificial intelligence are closely linked. The American philosopher J.R. Searle indicated that in the history of cognitive science, computers are key. Without digital computers, there would be no cognitive science (Baumgartner and Payr, 1995). It is particularly important in the research of face recognition and cognitive science. Whether people use face or not has a great relationship with their cognition, consciousness, psychology, and culture. The global workspace theory of Baars, a psychologist, posits that the brain is a modular information processing device composed of many neurons, and the information processing process is composed of different neurons with different divisions of labor and functions. The distributed operation process of specialized modules. The rapidly changing neuronal activity process constructs a virtual space called the global workspace at any given time through competition and cooperation between modules. Consciousness and unintentional state are generated through competition in the workspace. The generation of consciousness refers to all specialized modules in the brain responding to these new stimuli at the same time and analyzing and integrating this stimulus information in the global workspace through competition and cooperation until the best matching effect is achieved in the information processing between modules (Baars, 1988). Andrejevic and Volcic (2019) believes that exposing his face to the machine is in the interest of “efficiency” in this new world situation, creating contradictions with religious and cultural traditions. Face recognition largely depends on the exact meaning given to them by a wide range of actors, such as government, businesses, and civil society organizations (Norval and Prasopoulou, 2017).

Finally, there is the consideration of face recognition and privacy management. Governments and enterprises should strengthen the management and design of face recognition technology. The technology itself is neutral, and the intelligent measures developing from online to offline, as in the case of facial recognition, are targeted at efficient, convenient, and humanized services. Thus, the public must be willing to disclose their personal information to experience the benefits of the use of intelligent media entirely. As scholars have declared, “It is the default transaction rule in the data age to give up part of privacy for the fast operation” (Mao, 2019). Therefore, for the technology of face recognition at a crossroads, on the one hand, one cannot give up the application of technology because of privacy security. Instead, we should rely on smart hardware systems to empower cities and life with innovative technologies.

On the other hand, we cannot abuse this facial recognition technology after only viewing its bright prospects. Data security is always a crucial factor. Therefore, we believe that for face recognition technology, we must balance security, convenience, and privacy, strengthen the research on privacy issues in the field of big data networks, pay attention to the data flow behind it, constrain the technology with other evolving technologies, and cultivate the privacy literacy of the public.

## Data Availability Statement

The original contributions presented in the study are included in the article/supplementary material, further inquiries can be directed to the corresponding author/s.

## Ethics Statement

The studies involving human participants were reviewed and approved by the Secretariat of Academic Committee, Hangzhou Dianzi University. The participants provided their written informed consent to participate in this study.

## Author Contributions

TL and BY: conceptualization, software, and formal analysis. TL, SD, and YG: methodology and validation. TL and SD: investigation, resources, and data curation. TL, YG, and BY: writing—original draft preparation and visualization. TL, BY, and SD: writing—review and editing. All authors have read and agreed to the published version of the manuscript.

## Conflict of Interest

The authors declare that the research was conducted in the absence of any commercial or financial relationships that could be construed as a potential conflict of interest.

## Publisher’s Note

All claims expressed in this article are solely those of the authors and do not necessarily represent those of their affiliated organizations, or those of the publisher, the editors and the reviewers. Any product that may be evaluated in this article, or claim that may be made by its manufacturer, is not guaranteed or endorsed by the publisher.

## Footnotes

1
www.wjx.cn

## References

[B1] Andrejevic M. Volcic Z. (2019). “smart” cameras and the operational enclosure. *Telev. New Media* 22 1–17.

[B2] Baars B. J. (1988). *A Cognitive Theory of Consciousness.* Cambridge: Cambridge University Press.

[B3] Baumgartner P. Payr S. (1995). *Speaking Minds: interviews with Twenty Eminent Cognitive Scientists.* New Jersey: Princeton University Press. 204.

[B4] Bhattacherjee A. (2001). Understanding information systems continuance: an expectation-confirmation model. *Mis Q.* 25 351–370. 10.2307/3250921

[B5] Biometrics Identity Standardization [BIS] (2020). *2020 Face Recognition Industry Research Report.* Available online at: http://sc37.cesinet.com/view-0852f50939dd442daa42f566c950e336-fe654ac1ec464ae7b780f9fd78553c79.html [Accessed December 25, 2020]

[B6] Caputo F. Mazzoleni A. Pellicellic A. C. Muller J. (2020). Over the mask of innovation management in the world of Big Data. *J. Bus. Res.* 119 330–338. 10.1016/j.jbusres.2019.03.040

[B7] Caputo F. Scuotto V. Carayannis E. Cillo V. (2018). Intertwining the internet of things and consumers’ behaviour science: future promises for businesses. *Technol. Forecast. Soc. Change* 136 277–284. 10.1016/j.techfore.2018.03.019

[B8] Cha J. (2010). Factors affecting the frequency and amount of social networking site use: motivations, perceptions, and privacy concerns. *First Monday* 15 12–16. 10.5210/fm.v15i12.2889

[B9] Chen R. (2013). Living a private life in public social networks: an exploration of member self-disclosure. *Decis. Supp. Syst.* 55 661–668. 10.1016/j.dss.2012.12.003

[B10] Chen X. Y. Cliquet G. (2020). The blocking effect of privacy concerns in the “Quantified Self” movement–a case study of the adoption behavior of smart bracelet users. *Enterpr. Econ.* 4:109.

[B11] Chuah S. H. W. Rauschnabel P. A. Krey N. Nguyen B. Ramayah T. Lade S. (2016). Wearable technologies: the role of usefulness and visibility in smartwatch adoption. *Comp. Hum. Behav.* 65 276–284. 10.1016/j.chb.2016.07.047

[B12] Ciampi F. Demi S. Magrini A. Marzi G. Papa A. (2021). Exploring the impact of big data analytics capabilities on business model innovation: the mediating role of entrepreneurial orientation. *J. Bus. Res.* 123 1–13. 10.1016/j.jbusres.2020.09.023

[B13] Ciasullo M. V. Cosimato S. Pellicano M. (2017). Service Innovations in the Healthcare Service Ecosystem: a Case Study. *Systems* 5 2–19.

[B14] CNNIC (2020). *The 45th China Statistical Report on Internet Development.* Available online at: http://www.gov.cn/xinwen/2020-04/28/content_5506903.htm. [Accessed April 28, 2020]

[B15] Davis F. D. (1989). Perceived usefulness, perceived ease of use, and user acceptance of information technology. *Mis. Q.* 13 319–340. 10.2307/249008

[B16] Del Giudice M. Scuottoc V. Garcia-Perezd A. Messeni Petruzzellie A. (2019). Shifting wealth II in Chinese economy. the effect of the horizontal technology spillover for SEMs for international growth. *Technol. Forecast. Soc. Change* 145 307–316. 10.1016/j.techfore.2018.03.013

[B17] Del Giudice M. Scuottoc V. Orlando B. Mustilli M. (2021). Toward the human-Centered approach. A revised model of individual acceptance of AI. *Hum. Resour. Manag. Rev.* 100856. 10.1016/j.hrmr.2021.100856

[B18] Elias C. Francesco C. Del Giudice M. (2017). “Technology transfer as driver of smart growth: a quadruple/quintuple innovation framework approach,” *Proceedings of the 10th Annual Conference of the EuroMed Academy of Business* (Cyprus: EuroMed Press) 313–333.

[B19] Fletcher-Brown J. Carter D. Pereira V. Chandwani R. (2020). Mobile technology to give a resource-based knowledge management advantage to community health nurses in an emerging economies context. *J. Knowledge Manag.* 25 525–544. 10.1108/jkm-01-2020-0018

[B20] Fornell C. Larcker D. F. (1981). Evaluating structural equation models with unobservable variables and measurement error. *J. Mark. Res.* 18 39–50. 10.2307/3151312

[B21] Gefen D. Straub D. (2005). A practical guide to factorial validity using PLS-Graph: tutorial and annotated example. *Commun. Assoc. Inform. Syst.* 16 91–109.

[B22] Gerbing D. W. Anderson J. C. (1988). An updated paradigm for scale development incorporating unidimensionality and its assessment. *J. Market. Res.* 25 186–192. 10.1177/002224378802500207

[B23] Guo R. (2020). Face recognition, equal protection and contract society. *Ningbo Econ.* 02:42.

[B24] He J. P. Huang X. X. (2020). The smartphone use and eudaimonic well-being of urban elderly: based on intergenerational support and TAM. *J. Int. Commun.* 03 49–73.

[B25] Hichang C. (2010). Determinants of behavioral responses to online privacy: the effects of concern, risk beliefs, self-efficacy, and communication sources on self-protection strategies. *J. Inform. Privacy Secur.* 1 3–27. 10.1080/15536548.2010.10855879

[B26] Hsieh P. J. Lai H. M. (2020). Exploring people’s intentions to use the health passbook in self-management: an extension of the technology acceptance and health behavior theoretical perspectives in health literacy. *Technol. Forecast. Soc. Change* 161:120328. 10.1016/j.techfore.2020.120328

[B27] Jiang J. (2019). Infringement risks and control strategies on the application of face recognition technology. *Library Inform.* 5:59.

[B28] Joinson A. N. Reips U. D. Buchanan T. Schofield C. B. P. (2010). Privacy, trust, and self-disclosure online. *Hum. Comp. Interact.* 25 1–24. 10.1080/07370020903586662

[B29] Kalantari M. Rauschnabel P. (2018). “Exploring the Early Adopters of Augmented Reality Smart Glasses: the Case of Microsoft Hololens” in *Augmented Reality and Virtual Reality.* Ed Jung T. Tom Dieck M. (Germany: Springer). 229–245. 10.1007/978-3-319-64027-3_16

[B30] Kaur S. Gupta S. Singh S. K. Perano M. (2019). Organizational ambidexterity through global strategic partnerships: a cognitive computing perspective. *Technol. Forecast. Soc. Change* 145 43–54. 10.1016/j.techfore.2019.04.027

[B31] Khasawneh O. Y. (2018). Technophobia without boarders: the influence of technophobia and emotional intelligence on technology acceptance and the moderating influence of organizational climate. *Comp. Hum. Behav.* 88 210–218. 10.1016/j.chb.2018.07.007

[B32] Lee S. Y. Lee K. (2018). Factors that influence an individual’s intention to adopt a wearable healthcare device: the case of a wearable fitness tracker. *Technol. Forecast. Soc. Change* 129 154–163. 10.1016/j.techfore.2018.01.002

[B33] Lee Y. Kozar K. A. Larsen K. R. T. (2003). The technology acceptance model: past, present, and future. *Commun. Assoc. Inform. Syst.* 12 752–780.

[B34] Liu W. W. (2013). *Research on the Influence of Privacy Concerns on Users’ Intention to Use Mobile Payment.* Beijing: Beijing University of Posts and Telecommunications.

[B35] Lunney A. Cunningham N. R. Eastin M. S. (2016). Wearable fitness technology: a structural investigation into acceptance and perceived fitness outcomes. *Comp. Hum. Behav.* 65 114–120. 10.1016/j.chb.2016.08.007

[B36] Magni D. Scuotto V. Pezzi A. Del Giudice M. (2021). Employees’ acceptance of wearable devices: Towards a predictive model. *Technol. Forecast. Soc. Change* 172:121022. 10.1016/j.techfore.2021.121022

[B37] Mao Y. N. (2019). The first case of face recognition: what is the complaint? *Fangyuan Mag.* 24 14–17.

[B38] McKnight D. H. Lankton N. Tripp J. (2011). “Social Networking Information Disclosure and Continuance Intention: a Disconnect” in *2011 44th Hawaii International Conference on System Sciences (HICSS 2011).* (United States: IEEE).

[B39] Nasir S. Yurder Y. (2015). Consumers’ and physicians’ perceptions about high tech wearable health products. *Proc. Soc. Behav. Sci.* 195 1261–1267.

[B40] Nguyen T.-M. Malik A. (2021). Employee acceptance of online platforms for knowledge sharing: exploring differences in usage behavior. *J. Knowledge Manag.* Epub online ahead of print. 10.1108/JKM-06-2021-0420

[B41] Norberg P. A. Horne D. R. Horne D. A. (2007). The Privacy Paradox: personal Information Disclosure Intentions versus Behaviors. *J. Consum. Affairs* 41 100–126. 10.1111/j.1745-6606.2006.00070.x

[B42] Norval A. Prasopoulou E. (2017). Public faces? A critical exploration of the diffusion of face recognition technologies in online social network. *New Media Soc.* 4 637–654. 10.1177/1461444816688896

[B43] Nunnally J. C. Bernstein I. H. (1994). *Psychometric Theory.* New York: McGraw-Hill.

[B44] Papa A. Mital M. Pisano P. Del Giudice M. (2020). E-health and wellbeing monitoring using smart healthcare devices: an empirical investigation. *Technol. Forecast. Soc. Change* 153:119226. 10.1016/j.techfore.2018.02.018

[B45] Putnam R. D. (2001). *Making Democracy Work: civic Traditions in Modern Italy (trans. by Wang L & Lai H R).* Nanchang: Jiangxi People’s Publishing House. 195.

[B46] Qi K. P. Li Z. Z. (2018). A Study on Privacy Concerns of Chinese Public and Its Influencing Factors. *Sci. Soc.* 2 36–58.

[B47] Reyes-Mercado P. (2018). Adoption of fitness wearables: insights from Partial Least Squares and Qualitative Comparative Analysis. *J. Syst. Inform. Technol.* 20 103–127. 10.1108/jsit-04-2017-0025

[B48] Scherer R. Siddiq F. Tondeur J. (2019). The technology acceptance model (TAM): a meta-analytic structural equation modeling approach to explaining teachers’ adoption of digital technology in education. *Comp. Educ.* 128 13–35. 10.1016/j.compedu.2018.09.009

[B49] Simmel G. (2002). *Sociology: investigations on the Forms of Sociation (trans. by Lin R Y).* Beijing: Huaxia Publishing House. 244–275.

[B50] Turhan G. (2013). An assessment towards the acceptance of wearable technology to consumers in Turkey: the application to smart bra and t-shirt products. *J. Textile Inst.* 104 375–395. 10.1080/00405000.2012.736191

[B51] Venkatesh V. Davis F. D. (2000). A theoretical extension of the technology acceptance model: four longitudinal field studies. *Manag. Sci.* 46 186–204. 10.1287/mnsc.46.2.186.11926 19642375

[B52] Wang H. Lee M. K. O. Wang C. (1998). Consumer privacy concerns about Internet marketing. *Commun. ACM* 41 63–70. 10.1145/272287.272299

[B53] Wang Q. Sun X. (2016). Investigating gameplay intention of the elderly using an extended technology acceptance model (ETAM). *Technol. Forecast. Soc. Change* 107 59–68. 10.1016/j.techfore.2015.10.024

[B54] Werts C. E. Linn R. L. Jöreskog K. G. (1974). Intraclass reliability estimates: testing structural assumptions. *Educ. Psychol. Measur.* 34 25–33. 10.1177/001316447403400104

[B55] Xue K. He J. Yu M. Y. (2016). Research on Influencing Factors of Privacy Paradox in Social Media. *Contempor. Commun.* 1:5.

[B56] Yang H. Yu J. Zo H. Choi M. (2016). User acceptance of wearable devices: an extended perspective of perceived value. *Elemat. Inform.* 33 256–269.

[B57] Yu J. (2018). *Research on the Use Intention of VR Glasses Based on the Technology Acceptance Model.* Shenzhen: Shenzhen University.

[B58] Zahid A. Ajita R. (2017). A Face in any Form: new Challenges and Opportunities for Face Recognition Technology. *IEEE Comp.* 50 80–90. 10.1109/mc.2017.119

[B59] Zhang Q. J. Gong H. S. (2018). An Empirical Study on Users Behavioral Intention of Face Identification Mobile Payment. *Theor. Pract. Fin. Econom.* 5 109–115.

[B60] Zhang X. J. Li Z. Z. (2018). Research on the Influence of Privacy Concern on Smartphone Users’ Behavior Intention in Information Security. *Inform. Stud. Theor. Appl.* 2 77–78.

